# Case series on Zika virus genome detection in cerebrospinal fluid samples from newborns at a Brazilian tertiary hospital

**DOI:** 10.1590/S1678-9946202668050

**Published:** 2026-08-03

**Authors:** Suzana Ferreira Zimmerman, Jamil Pedro de Siqueira Caldas, Rodrigo Gonçalves de Lima, Sandra Helena Alves Bonon, Plínio Trabasso, Sergio Tadeu Martins Marba

**Affiliations:** 1 Universidade Estadual de Campinas, Faculdade de Ciências Médicas, Departamento de Pediatria, Campinas, São Paulo, Brazil; 2 Universidade Estadual de Campinas, Faculdade de Ciências Médicas, Laboratório de Virologia, Campinas, São Paulo, Brazil; 3 Universidade Estadual de Campinas, Faculdade de Ciências Médicas¸ Departamento de Clínica Médica, Campinas, São Paulo, Brazil

**Keywords:** Newborn, Zika virus infection, Congenital Zika syndrome, Polymerase chain reaction, Cerebrospinal fluid.

## Abstract

Detection of ribonucleic acid (RNA) Zika virus in cerebrospinal fluid (CSF) by molecular methods serves as a marker of the involvement of congenital infection in newborns’ central nervous system. This study aims to describe the detection of Zika virus genome in CSF samples from newborns (aged from one to 28 days) using molecular biology and its clinical consequences following the Zika virus epidemic from October 2015 to May 2017, in Brazil. This prospective descriptive study molecularly analyzed 151 CSF samples from neonates at Professor Doutor Jose Aristodemo Pinotti Hospital, collected for cytological and biochemical diagnosis from June 2017 to June 2021. After the consent of legal guardians, signing of free and informed consent forms, and the issuance of these results, the remaining CSF was sent to the Virology Laboratory of School of Medical Sciences in the State University of Campinas (Unicamp) and was subjected to RNA extraction and real-time polymerase chain reaction (RT-PCR). A review of patients’ medical records and descriptive statistics were also performed. This study obtained 151 CSF samples, four of which were PCR positive for Zika virus (2.6%, with two presenting congenital malformations). These results suggest the clinical relevance of etiological diagnosis of congenital Zika syndrome by molecular biology in newborns’ CSF samples even after the Zika virus epidemic (which occurred from October 2015 to May 2017) in Brazil.

## INTRODUCTION

The Zika virus is an arbovirus that belongs to the Flaviviridae family. This enveloped virus has a ribonucleic acid (RNA) genome^1^ that causes infections that are transmitted by arthropod vectors in some regions of the planet, primarily tropical climates^2^ ^-^ ^4^, such as Brazil. Most individuals infected with the Zika virus are asymptomatic, but the disease often causes nervous system infections^5^. The consequences of these infections (acquired by vertical transmission) include stillbirths, prematurity, uterine growth restriction, and congenital malformations^6^ ^-^ ^8^.

The Zika virus epidemic occurred from October 2015 to May 2017 in Brazil^6^. Several studies have described the symptoms and signs associated with this virus, particularly the association between Zika virus and a congenital syndrome in newborns-described in the literature since 2016, highlighting the numerous cases of microcephaly in neonates^7^ ^),(^ ^8^. Furthermore, other possible malformations may be associated with patients with positive cerebrospinal fluid (CSF) tests for this pathogen^7^ ^-^ ^9^. Detection of RNA virus in CSF by molecular methods, such as real-time polymerase chain reaction (RT-PCR), serves as a marker of the involvement of congenital infection in the nervous system^10^.

This study describes-following the epidemic-cases of newborns with signs that may be associated with this virus and are part of the congenital syndrome, with other malformations in addition to microcephaly^10^.

The risk of transmission from mother to fetus depends on the type of infection and time of gestation. The clinical signs are often nonspecific or even absent, requiring an efficient diagnostic method for rapid detection. Furthermore, diagnosing congenital infections based on maternal serological result is difficult because the infection may not have generated an immune response in the body. Therefore, the development of diagnostic methods with greater accuracy, sensitivity, and specificity to detect congenital infections is essential, emphasizing the relevance of molecular biology^10^. 

This study aims to describe the detection of Zika virus genome in CSF of newborns aged from one to 28 days and the clinical signs (congenital malformations) in these neonates using molecular methodology and the evaluation of these newborns’ medical records, even after the Zika virus epidemic (which occurred from October 2015 to May 2017) in Brazil.

### Ethics

This study neither requested additional collections nor offered harm to patients. It was approved by the Ethics Committee on Research in Human Beings following the standards outlined in Resolution Nº 466/2012 of the National Health Council via Plataforma Brasil under Certificate of Presentation for Ethical Review (CAAE Nº 86760218.3.0000.5404).

## MATERIALS AND METHODS

This was a prospective descriptive study: CSF samples from neonates served at the Neonatal Unit of the Doutor Jose Aristodemo Pinotti Hospital in the State University of Campinas (Unicamp), Sao Paulo State, Brazil (at latitude: 22°54’20.16” South and longitude: 47°03’38.88” West) were collected from June 1, 2017, to June 30, 2021, and analyzed.

Newborns (aged from one to 28 days) admitted at the Neonatal Unit of that hospital were included in this study. Their CSF was collected for cytological and biochemical tests. The samples were sequentially selected based on the available remaining CSF samples in the Biological Fluids Laboratory of Clinical Pathology Department at Hospital de Clinicas at Unicamp. Patients with insufficient sample volume (less than 400 microliters) for analysis and those older than 28 days were excluded from this study.

After the samples had been sent to the Laboratory of Biological Fluids of the Department of Clinical Pathology at Hospital de Clinicas at Unicamp for the cytological and biochemical tests required for the clinical conduction of cases, the remaining CSF was stored in a freezer at −20 °C. After these results were issued, informed consent forms were signed by the newborns’ parents or legal guardians before the PCR tests. Then, the CSF samples were sent to the Virology Laboratory of the School of Medical Science, Unicamp, in which they were stored in a freezer at −80 °C.

Once thawed, the CSF was subjected to RNA extraction. Next, the presence of Zika virus was assessed by RT-PCR.

RNA was extracted from 140 microliters of the CSF sample using a Biogene Bioclin viral DNA/RNA extraction instrument (Quibasa, Belo Horizonte, Minas Gerais, Brazil), following the manufacturer’s instructions. The final obtained volume totaled 200 microliters. This kit has been validated for use with CSF samples^7^ ^),(^ ^8^.

For the reverse transcription reaction on the extracted RNA samples, the Access Quick™ RT-PCR System kit (Promega) was used. The mixture consisted of five microliters of RNA, one unit of reverse transcriptase enzyme (Promega, USA), 12.5 microliters of Access Quick™ Master Mix, one microliter of Random primer, 20 units of ribonuclease inhibitor (Rnase OUT-Invitrogen, USA), and enough water to make 20 microliters. The mixture containing the RNA and enzyme was incubated at 72 °C for 5 min. After incubation, the mixture was placed in an ice bath, in which the master mix was added to it, followed by a second incubation for 60 min at 45 °C. Then, the complementary DNA was obtained and stored at −20 °C. To monitor the presence of inhibitors, Beta2-microglobulin gene primers (F: 95GGTGTCTTGAGGCTCAGGGAG; R: CAACTTCAATGTCAATGTCGATGGATG) were included in each sample as a control. RNA extraction was repeated when the PCR samples for the Beta2-microglobulin gene were negative.

For the Zika virus, the following primers (oligonucleotides) were used: F: CCGCTGCCCAACACAAG; R: CCACTAACGTTCTTTTGCAGACAT;

Probe: FAM - AGCCTACCTTGACAAGCAGTCAGACACTCAA (Lanciotti *et al.* ^11^).

RT-PCR was performed using TaqMan™ Fast Advanced (Applied Biosystem), primers, and hydrolysis probes (sequences described in the literature)^11^, synthesized as PrimeTime qPCR Primers (Integrated DNA Technologies Incorporated, USA). The tests were performed on the Step One Plus™ Real-Time PCR System (Applied Biosystem) under the following conditions: 95 °C by 20 s, 40 cycles of 95 °C at 1 s, and 60 °C for 20 s. 

This qualitative study aimed to find the studied genome in a CSF sample. The possibility of cross-reaction was reduced due to the specificity of the used method (based on primers designed specifically for Zika virus).

These four cases found were also tested for other important causes of congenital infection via PCR for other pathogens, such as toxoplasmosis, cytomegalovirus, herpes simplex types 1 and 2, Varicella zoster virus, Epstein-Barr virus, and Parvovirus B-19. 

Patient data were obtained from the hospital systems (intranet) and the Management Application for University Hospitals, which offer patients’ information, exams’ results, diagnostic hypotheses.

The following variables were collected from the newborns’ medical records: gender, age, gestational history of the mother, and neonatal antecedents (weight, head circumference, and length at birth; gestational age, Apgar score at first and fifth minutes, anomalies, and presence or absence of microcephaly and any other malformations), as were the corresponding complementary tests and clinical evolution: initial diagnostic hypothesis, cytological and biochemical results of routine CSF exams (with protein, glucose, red blood cell, and leukocyte counts), and bacterioscopy and culture of CSF samples.

The following were considered in the evaluation of the CSF cytology and biochemistry results: hyperpro­teinorrhachia above 42 milligrams per deciliter and pleocytosis (leukocytes above 15 cells per milimiter^3^). In the case of CSF containing red blood cells (probably due to traumatic puncture), the ratio of one leukocyte for every 500 red blood cells was ignored, as were one mg for every 500 red blood cells in the case of proteinorrhachia. CSF glucose levels were analyzed in relation to blood glucose levels (considered hypoglycorrhachia if below 66% of serum glucose).

Measures of central tendency (means, medians, and frequencies) were used in the descriptive statistics. The data were analyzed on Statistical Package for the Social Sciences (SPSS) (version 16.0, SPSS Incorporated, Chicago, IL, USA) and Stata (version 12.0, StataCorp, College Station, TX, USA).

## RESULTS

### Case series report

This study obtained 151 CSF samples from newborns, four of which (2.6%) were positive for Zika virus. This research detected the RNA from this pathogen in the newborns’ CSF via RT-PCR. All samples had negatives bacterioscopy and culture.

The mothers of these four newborns (described below) underwent no testing for Zika virus during prenatal care. All four mothers lived at the metropolitan region of Campinas, Sao Paulo State, Brazil, and none of them had traveled during pregnancy.

Table 1 describes the characteristics of the CSF and of the neonates in the positive cases for the Zika virus genome.

### Case One

A 17-day-old female patient received an initial diagnosis of sepsis. Therefore, a CSF sample was collected in March 2018, which showed hyperproteinorrhachia, hypoglycorrhachia, and pleocytosis. Furthermore, RT-PCR found the Zika virus genome. However, this patient had no apparent central nervous system (CNS) malformations or microcephaly.

**Table 1:** Epidemiological characteristics of newborns, year of CSF collection, cytology and biochemistry analysis, RT-PCR for Zika virus in CSF samples, and assessment of congenital malformations

Patient’s gender	Age	Year of CSF collection	Leukocytes count	Proteins	Glucose	RT-PCR	Congenital malformations
Female	17 days	2018	Increased	Increased	Diminished	Positive	None
Female	2 days	2018	Normal	Increased	Diminished	Positive	Hydrocephalus
Female	2 days	2018	Normal	Normal	Normal	Positive	None
Male	13 days	2019	Increased	Increased	Normal	Positive	Spina bifida, lumbosacral myelomeningocele, Arnold-Chiari syndrome type II, and hydrocephalus

RT-PCR = Real-time PCR; hyperproteinorrhachia > 42mg/dL; pleocytosis (leukocytes >15 cells/mm3); hypoglycorrhachia: below 66% of the serum glucose.

### Case Two

A two-day-old female patient had hydrocephalus since birth. Thus, a CSF sample was collected in April 2018, showing hyperproteinorrachia, hypoglycorrhachia, and normal cellularity. Furthermore, RT-PCR found the Zika virus genome.

### Case Three

A two-day-old female neonate received an initial diagnosis of sepsis. Therefore, a CSF sample was collected in October 2018. It had the following characteristics: no alteration in cellularity or biochemistry but positive detection for Zika virus via RT-PCR. This patient had no apparent CNS malformations or microcephaly.

### Case Four

A 13-day-old male patient was born with spina bifida, ruptured lumbosacral myelomeningocele, Arnold-Chiari syndrome type II, and hydrocephalus. He underwent CSF puncture in May 2019. The sample showed hyperproteinorrhachia, pleocytosis, and normal glucose level. It tested positive for Zika virus (RT-PCR).

All these patients were full-term newborns and weighed more than 2.5 kilograms at birth. The descriptive statistical analysis showed a median age of 7.5 days and a mean age of 8.5 days at the time of lumbar puncture for CSF collection. Furthermore, 75% of the newborns were female.

## DISCUSSION

The risk of transmission from mother to fetus depends on the pathogen and the trimester of gestation. Generally, the most severe conditions emerge in the first trimester of pregnancy^4^ ^-^ ^6^. 

Araújo *et al*.^7^ ^),(^ ^8^ published a case-control study in 2016 and 2017 (conducted in the Pernambuco State, Brazil) that proposed the association between microcephaly and congenital Zika virus infection. They also reported evidence of the lack of effect of other potential factors, such as exposure to the insecticide pyriproxyfen or vaccines during pregnancy. In these studies, mothers of newborns with microcephaly had more serological markers of previous Zika virus infection than those of controls (without microcephaly), although mothers in both groups were PCR negative^7^ ^),(^ ^8^. Among newborns, 36% of the microcephaly cases and none of the 173 of the control infants had laboratory-confirmed Zika virus infections. Laboratory and imaging were available for 87% of the cases: among 23 patients with positive PCR, 43% tested positive for Zika virus and had brain abnormalities (as in two of the four cases in this study); 13 were positive for Zika virus infection but had no brain abnormalities, whereas 20% were negative for Zika virus but had brain abnormalities. That study reinforced the association between microcephaly and congenital Zika virus infection^8^. 

In another study in that same year^9^, during the Zika epidemic in French Guiana, positive PCR for this virus occurred in 57% of newborns’ CSF (validating PCR as a technique for CSF samples prior to this study). Maternal-fetal transmission occurred in 25% of exposed fetuses, being associated with poor early fetal and neonatal outcomes-fetal loss or severe complications of congenital Zika syndrome-in 33.3% of infected fetuses.

The literature links the epidemic of microcephaly to maternal Zika virus infections during gestation^12^, with evidence of congenital infections (calcifications, ventriculomegaly, and cortical developmental disorder) and ruling out the other main causes of congenital infection (such as cytomegalovirus and toxoplasmosis) and other genetic or environmental causes^13^ ^-^ ^16^. Likewise, this study, conducted at Professor Doutor Jose Aristodemo Pinotti Hospital, ruled out the other main agents that cause congenital infections, such as cytomegalovirus, herpes simplex types 1 and 2, Epstein-Barr virus, Varicella zoster virus, and toxoplasmosis.

The World Health Organization defines microcephaly as a head circumference equal to or below 31.9 cm for boys and equal to or below 31.5 cm for girls born at term^12^.

The microcephaly epidemic had grown considerably in the second half of 2015. Brazil confirmed 2,205 cases by the end of 2016. In 2017, the country recorded new cases of Zika virus infection, with an incidence rate of 1.9 cases per 100.000 inhabitants^11^.

The most likely cause of the head circumference reduction is related to the presence of the virus in the CNS, directly promoting neuronal death or activating the immune responses of infected fetuses, compromising the structure and function of important areas^17^ ^-^ ^19^.

The Brazilian Ministry of Health declared the end of the public health emergency of national concern in May 2017 due to the decrease in Zika and microcephaly cases throughout Brazil^6^. However, the end of the emergency does not mean the end of surveillance and care, as per the four cases in this study (Results section).

In short, the identification of Zika virus as a CNS pathogenic agent via PCR in CSF in recent studies^19^ ^),(^ ^20^ supports the concept that this pathogen remains an important infectious cause of microcephaly and other congenital malformations, even after the declared end of its epidemic (which occurred from 2015 to May 2017) in Brazil.

### Limitations

This study has some limitations that are expected in a case series: causal and clinical significance of Zika virus detection in CSF is limited in light of the small number of positive cases and the descriptive nature of the study.

On the other hand, this study is valuable in describing positive cases of Zika virus in newborns’ CNS even after the end of the epidemic, showing that the virus still circulates in Sao Paulo State, Brazil. However, we must also consider that the causal relationship between the vertical transmission of the Zika virus and the presence of congenital malformations in the CNS had already been well established in the literature.

## CONCLUSION

The evaluation of medical records in some patients whose CSF samples tested positive for the molecular detection of Zika virus genome found congenital malformations and alterations in CSF cytology and biochemistry, suggesting the clinical relevance of etiological diagnosis of congenital Zika syndrome via molecular biology newborns’ CSF even after the Zika epidemic (which occurred from October 2015 to May 2017) in Brazil. The Zika virus continues to circulate and cause infections in newborns in the Sao Paulo State, even though the World Health Organization declared the end of the epidemic in Brazil in May 2017.

## Data Availability

The Authors Will Make No Additional Data Available Due To Ethical Restrictions Related To Patient Confidentiality.
